# Observations on the Poisonous Properties of Sulphate of Quinine

**Published:** 1847-11

**Authors:** 


### BIBLIOGRAPHICAL NOTICES.

---

ART. VI.—*Observations on the Poisonous Properties of the Sulphate of Quinine.* By WILLIAM O. BALDWIN, M.D., of Montgomery, Ala. From the American Journal of the Medical Sciences, April, 1847.

The object of the author of this pamphlet is to awaken the attention of the profession to the dangers of a practice become very prevalent in many parts of our country. He holds sulphate of quinine to be poisonous in certain doses, and as such warns physicians against its too liberal administration. If correct in these apprehensions, he has performed an opportune service to science and humanity, for most certainly very few practitioners participate in his fears. We are prepared to learn that his warning voice has been listened to with general incredulity, if not treated with derision. Men who have given quinine in doses of a drachm, and witnessed no untoward effects from such quantities, will laugh at the idea that it is a poison. And, in truth, we must say, that we were ourselves startled by the proposition. So familiar had we grown with this heroic practice, that we had come to regard it as quite harmless, but, at the same time, let us add, as quite unnecessary in a great majority of the cases where it is resorted to.

Ultraism is inseparable from human nature, and in no human pursuit is it exhibited more forcibly than in medicine, the history of which presents us with endless extravagances. A new remedy is introduced, and forthwith it is extolled as a panacea, to be decried in due time as a poison. It is found good in some cases, and must therefore be a catholicon; it has cured some diseases when administered in small doses; if given in larger, it would doubtless cure more. The reasoning is not sound, and leads to bad practice. We are not yet ready to admit the truth of Dr. Balwin's conclusions, but we are satisfied that he has attacked an indefensible practice. Whether quinine be poisonous or not, it is given in doses wastefully large, and such a practice ought to be condemned.

About fifteen years ago, Dr. Fearn, of Alabama, administered quinine to his patients, in a malignant form of fever, in quantities much larger than had been the custon of the profession. The course was found to succeed well, and thenceforth the practice spread among his brethren in Alabama. Communications were published in the Transylvania Journal of Medicine—among the rest, one by Dr. Thomas H. Todd, now of Memphis, Tenn.—insisting upon the efficacy of large doses of the remedy in intermittent, and especially in congestive fever. The tide, which has rolled on ever since in favor of the practice, the author of the paper before us is disposed to check. We most cheerfully yield him the necessary space to exhibit the evidence upon which his convictions rest, and, as an important element of this—the "basis" of his belief, as he terms it—quote the following case:

"*Case of Convulsions, Blindness, and Death—the Effect of the Sulphate of Quinine.*—On the 2d day of July, 1846, I was called to see 'Sally Ann,' a little negro girl, aged about six years, the property of H. M. Elmore, Esq., living five miles from this place. This is the fifth day of her disease. Mr. E. states that her fever has been higher on alternate days (tertian remittent), pulse rising to about 160 on the days of the exacerbation of fever, and falling to about 120 on the succeeding days, the exacerbation commencing early in the morning and declining in the latter part of the succeeding night. He has given her some mild cathartics, and in one of her remissions he gave her quinine. This is the day for her exacerbation of fever to take place. Pulse now 130; skin hot and dry; tongue moist, and covered with a thick dark fur; abdomen somewhat distended and tender on pressure; complains of pain in the head, and thirst. ℞. Venesect. ad ℥iv or ℥vj; submur. hyd. grs. ij; p. opii gr. 1-6; M. To be taken and repeated at intervals of three hours; warm bath, mustard poultice to abdomen.

mucilaginous drinks. Directed Mr. E. to repeat the bleeding if her fever should rise in the course of the day.

"3d. Fever again yesterday; pulse rose to 160; bled again. Medicine produced two small, dark, thin evacuations, interspersed with mucus. Pulse this morning 130; condition in other respects about as it was yesterday. ℞. Sulph. quin. gr. ij, to be given and repeated every second hour; other prescriptions to be continued.

"4th, 8 o'clock, a. m. Pulse 132 (did not rise over that yesterday); *countenance anxious; irregular and heavy breathing; extreme restlessness*, constantly rolling her head from side to side, and frequently altering the position of her body with great quickness of motion. Had two motions, dark and rather more consistent, containing some mucus and two small round worms; condition in other respects the same.

"This is the day for her exacerbation of fever to come on, which heretofore commenced rising about 8 o'clock. I remained with her until 12 o'clock, (during which she did not take any more quinine), at which time her condition remained the same, except not quite so restless. Looking upon this restlessness, constant motion of the head, tossing of the body, etc., as an indication that the quinine was exerting an unfavorable influence, I determined to withdraw it, but to do so gradually, and in such a manner as to extend its influence through that day; consequently ordered the same quantity to be given at intervals of four hours, and to be suspended altogether after 4 o'clock in the morning. Supposing I had discovered slight symptoms of ptyalism, ordered calomel and opium powders to be discontinued. ℞. Mush poultice and mucilaginous drinks.

"5th. Pulse 128 to 130. *Exacerbation of fever did not come on yesterday;* last quinine was taken at 4 o'clock this morning. Except a slight abatement of the '*restlessness*,' etc., condition pretty much as it has been for the last several mornings; one motion from bowels, same character; no symptoms of ptyalism this morning. ℞. Calomel and opium resumed in same doses; to be enveloped in a poultice towards the middle of the day; mucilaginous drinks.

"On returning next morning I found her dead. Mr. Elmore stated that he put her in a poultice as I had directed, and that it had a fine effect, producing a free, warm and general diaphoresis, which lasted three or four hours. On discovering this, he concluded she was in a 'fine condition' for taking quinine, and soon after (2 o'clock, p. m.) gave her four grains, and, in the course of three hours after, four grains more.

Shortly after he gave her the last dose her skin became dry again, succeeded by restlessness. About 6 o'clock she had a convulsion. After this he noticed that the pupils of *her eyes were dilated*, and soon discovered she was *totally blind.* When asked if she knew her mother and other persons who were placed before her in a bright light, her eyes would wander about, she apparently endeavoring to fix them on some object, and then she would reply, 'I can't see them.' The *dilatation of the pnpils, blindness, restlessness, convulsions, etc.*, continued until 8 o'clock, when she died. The convulsions were described by Mr. E. as being of a most violent character; but notwithstanding she retained in the intervals perfect possession of her mental faculties, and an unusual degree of pertness for children of her age.

"I was not prepared to make a thorough post mortem examination, and therefore made a partial one only of the stomach and bowels. Found considerable vascularity in portions of the small intestines and stomach, the former containing secretions of a yellowish and greenish substance, intimately blended with mucus; no worms; pupils enormously dilated."

Dr. B. goes on to comment thus upon the foregoing:

"A review of this case leaves no doubt upon my mind of the direct agency of the quinine in producing death. The quantity given immediately before death (8 grs.) would not of itself, I am disposed to think, have produced the fatal result, separate from the agency of that which had been given previously; but at the time these last portions were given, it must be remembered that the system was still charged with the quinine to some extent, for up to four o'clock that morning it had been regularly introduced into the stomach, at intervals, for nearly two days. The accession of fever which should have taken place on the 4th was prevented. Now, it is very sure that the patient either died from the effects of the quinine, or that the paroxysm of fever, which had been arrested or suspended on the 4th, came on on the 5th and killed her. The latter could not have been the case, for we find her an hour or two before she commenced taking the quinine (the second time) in a warm, free and diffused perspiration. The most conclusive evidence, however, to my mind, that the quinine *did* kill the patient, is the characteristic train of symptoms which immediately followed its administration, and preceded death—the extreme restlessness, dilatation of the pupils, blindness, and convulsions. The exacerbating feature of the disease had been broken up, after which there was nothing to forbid the hope of her recovery, and, apart from the effects of the quinine, there was certainly nothing in her condition to account for her death *at that time.*"

He cites the following case, furnished by the experience of a professional friend:

"A man about 30 years old, habits and constitution good, 'has had a cold for five or six days.' On the morning of the 25th of October, 1843, was taken with a chill, the sensation of coldness continuing nearly all day, with a severe and acute pain in the right side, frequent cough, and severe headache.

"26th. Pulse 108, rather soft, and moderately full; skin hot; cough frequent; expectoration scant and fluid, slightly tinged with blood; severe headache, and great turgidity of the veins about the forehead; tongue nearly natural; bowels rather costive; thirst; slight dullness on percussion over lower part of right lung, and nearly over the whole lung the natural respiratory murmur is replaced by an irregular undulatory sound, resembling somewhat that produced by the application of a 'sea-shell' to the ear. In the left lung the sounds are healthy. Eyes very much injected; slight delirium. ℞. Calomel grs. v; p. opii gr. ¼. M. Repeat every third hour; mucilaginous drinks.

"Noon. Pulse 115; has taken three pills; expectoration of a reddish orange; otherwise same. ℞. Antim. tart. grs. ii; aqua pura ℥iv; m.; ℥ss every hour; ℥iv blood from the frontal vein.

"Vesper. Pulse 120, full and soft; respiration 36; dullness extending upwards; no sound present over dull part except a slight friction sound, more audible during inspiration; over sonorous part of lung, on applying stethoscope, only the undulatory sound heard, respiratory murmur being absent; cough frequent and attended with great pain; expectoration same; tongue moist; collection of sordes on upper teeth; breath fetid; quite delirious, talking incoherently, and is anxious to get up and leave the bed; complains much of head and side. Took four successive doses of medicine, but vomited a large quantity of bile just before the time to take the last dose. ℞. Continue antimonial every hour.

"8, p. m. Pulse 104; respiration 28; sweating very freely; says he feels easier since he had a large, thin, yellow operation, about half an hour ago.

"10, p. m. Sleeps apparently easy for a few minutes, and then awakes and talks deliriously; pulse 98, full and soft; respiration 26; tongue rather disposed to be dry on dorsum; skin moist; complains of sharp pains darting through head; side easier. ℞. Continue antimonial every third hour; quinine grs. xij at 11 o'clock, grs. viij at 2 o'clock, and grs. viij at 5 o'clock in the morning.

"27th. Pulse 104, more firm; respiration 28; skin became dry at a quarter after 10 o'clock last night, and so continues; continued to have short naps, between which he would be awake and delirious; had three thin dark evacuations; after the second he took fifteen drops tr. opii, same quantity after third. He missed one dose quinine (viij grs.) last night; complains much of the same darting pains through head and side; took about ℥xij blood, from which he experienced no immediate relief of pain; pulse softened a little under it, the frequency remaining the same; no nausea; a little deaf; cough suppressed. ℞. Now, 7 o'clock, take quin. grs. viij. Antimonial mixture, $\frac{1}{4}$ gr., every hour; tinct. opii gtt. x after each evacuation.

"Noon. Pulse 100, softer; respiration same; skin moist; one more evacuation; sleeps steadily now, except when he is awakened to take medicine. ℞. Continue same.

"3, p. m. Asleep, and sleeps soundly nearly all the time, except when awakened to take medicine; pulse 100, full; respiration 28; skin hot and dry; tongue moist and white; cough frequent; expectoration scant, opaque, and without blood; slightly deaf; nausea. ℞. Continue same.

"Vesper, 5, p. m. Is scarcely at all delirious; forehead moist; thirst; complains much of weakness, emptiness and nausea; pulse 98; respiration 24; sleeps well; has been up several times, but no evacuation. ℞. Give quinine grs. viij; continue antimonial.

"8, p. m. Pulse 98; respiration 20; otherwise same. ℞. Quin. grs. xxxvj, calomel grs. xij, tart. emetic grs. iss, p. opii gr. j; m.; in pls. no. xviij div.; three every second hour; continue antimonial, gum-water, etc.

"28th. Pulse 85; respiration 20; one evacuation; less dullness; expectoration easy and free, opaque and yellowish; tongue moist and white. ℞. Continue same, leaving tartar emetic out of pills, and instead ℞. antim. tart. grs. iij, water ℥iv; m.; ℥ss every hour.

"Noon. Pulse 98; respiration 26; skin hot; expectoration more difficult and scant; has just thrown up some bile; considerable nausea; an orange-colored matter deposited on the teeth, at the junction between them and the gums. ℞. Continue same, omitting one dose of the antimonial solution.

"3, p. m. Called to see him. He had a little while before been taken with a *jerking motion* of the whole body, which lasted several minutes, and immediately after his *vision was so imperfect* that he could scarcely distinguish anything. When I arrived, whole surface hot; respiration irregular, from 11 to 20; pulse 100, full and rather firm; temporal veins turgid, and temporal arteries throbbing; *great restlessness*, anxiety and alarm; thirst increased; tongue more dry, white and rough; *pupils dilated;* he dozes two or three minutes at a time, then starts up, breathing more quickly and audibly; cough frequent and dry; respiratory murmur heard over the greater extent of lung. The convulsive movements of body came on every ten or twelve minutes, sometimes apparently of the whole body, at others confined to the arms; in the latter case, by grasping the arms with moderate firmness, or pressing them against the bed, the motions could be arrested immediately. He was not insensible during the convulsions, nor was there foaming at the mouth, but occasionally a vacant and staring look, and rolling up of the eyes. By half after four o'clock *he was completely blind.* On drawing (4½ o'clock) an ounce of blood from a small orifice, it jetted out with force, and was the color of arterial blood, and much buffed when cold. From half past four o'clock till six he had but one slight convulsion. After taking an ounce of blood, I thought the pulse softened, but regained its same character by the time I had tied the arm."

Dr. Baldwin entered upon the use of quinine without any prejudices against the remedy, and was liberal in the application of it to febrile diseases. Like his brethren in the same region, he was even disposed, as he now concludes, to exaggerate its efficacy in mammoth doses. He adds:

"Notwithstanding this acknowledged partiality for it, I have never administered it in the enormous doses prescribed by those who boast of having given their thirty grains repeated every half hour until two hundred and forty grains were introduced into the system, or their several ounces in the treatment of one case of fever. I have very rarely given more than ten grains at a single dose; my usual mode having been to give from four to six grains, and repeat at intervals of two, three, or four hours, until twenty-four or thirty-six grains had been introduced into the system, in adults. Occasionally I

have given it in larger doses, but these cases have been extremely rare, believing that the quantities specified above, given in one intermission or remission at the proper periods, and aided by suitable auxiliaries, could accomplish ordinarily all that the remedy was capable of, and these enormous doses, to say the least of them, unnecessary. Sometimes, owing to want of time, I have given from twenty to twenty-four grains at one dose, but in such cases have never repeated it in the same dose.

"Second to none other, quinine must ever hold the first rank in our materia medica in the treatment of southern diseases. I am accustomed to witness its peculiar and almost wonderful influence in arresting certain forms of diseases; but yet that it has gone far beyond its proper bounds, and is now being used too recklessly and indiscriminately, I feel most fully persuaded, and that it may be directly and fatally poisonous I am entirely convinced. Yet I doubt not many an enthusiast of the 'Dr. Bazire' order will smile indignantly at thus seeing such properties attributed to his favorite and 'harmless' remedy.

"To show how completely the world has run riot upon this subject, we quote the following remarks by Dr. Dickson, in an article in the Southern Journal of Medicine, vol. i, no. 1:

"'A medical friend in Alabama assures us that he had administered thirty grains of the solution of quinine every hour for seventeen successive hours; and we have heard authentically of a western physician who emptied into the stomach of a patient, laboring under bilious remittent, an ounce bottle of sulphate of quinine in one night. From thirty to fifty grains are now spoken of as not unfamiliar doses, and even one hundred grains are occasionally given at once, and, we are assured, both with safety and striking success. It is in France, however, that the largest amounts of this salt have been employed. Guersent and Reveillon speak of a Dr. Bazire as having been remarkable for his enthusiastic and exclusive confidence in quinine as a remedy for the violent and pernicious intermittents of the department in which he practiced. Madame Bazire being seized with the prevailing malaria fever, took from him, 'in a very short space of time,' two hundred and forty grains (sixteen grammes) of sulphate of quinine. Soon after, the symptoms increasing, he gave her at one dose three hundred and seventy-five grains (twenty-five grammes), more than three-fourths of an ounce. At this juncture, fortunately for her, he fell sick, and the care of her devolved on other hands. He hastened to administer to himself, by the mouth and by the rectum, nine hundred grains of the sulphate of quinine (sixty grammes), nearly two ounces, in a very short space of time; and still 'further took, during the space of eight or nine days, five ounces.' He died a martyr to his reckless and implicit reliance on the drug; and she recovered imperfectly, having been for a long time both deaf and blind, the senses both of sight and hearing still remaining feeble.'

"In the same article, Dr. Dickson—although in speaking of the experiments of Melier, and in other places in the article just referred to, he expresses his fears of too large or 'misapplied' doses of this drug becoming 'dangerous and destructive,' and is too cautious and prudent to give these heroic doses himself—yet says: 'But Madame Bazire took a dose of three hundred and seventy-five grains, with something less than fatal effect—an experiment not likely to be repeated by any practitioner in his senses. There seems, then, to be no risk of mortal error in regard to mere quantity, or next to none.' How far Madame Bazire was indebted for her lucky escape to the fortunate rejection of the medicine by the stomach, or how much of the alkaloid was rendered inert by the want of a solvent, or how far its action was modified by the engrossing nature and extent of the preoccupying morbid action which may have been present in the system at the time, we are only eft to conjecture. We are not told, either, how much of this quantity was given by the rectum, which would render its action less powerful. This, however, seems to have been a favorite mode of giving it with Dr. Bazire, and, indeed, one might suppose an ordinary stomach scarcely capacious enough to receive his enormous doses. But all this as it may be, we do know that other unfortunate individuals, to whom, perhaps, the fates were less propitious than to Madame Bazire, have fallen victims to infinitely smaller doses. M. Briquet reports two fatal cases, in one of which death occurred after a 'comparatively small quantity of quinine' had been taken; in the other, about one hundred grains, given during the space of two days; and in another case, which occurred in the practice of M. Recamier, death occurred after the administration of nearly ℥ij in doses of five grains repeated every hour."

Dr. Baldwin thus sums up:

"From all that I can gather, I am disposed to think from fifty to eighty grains of a pure article of quinine, given in solution at one dose, will produce death nine times out of ten in healthy adults, and occasionally even smaller quantities. How far its operation may be modified by morbid action is a matter for consideration at the bedside.

"I will not accuse any member of the profession of a want of common honesty in not reporting the fatal cases from the poisonous effects of quinine which may have occurred under his observation; but all the circumstances constrain me to believe that many results of this kind have occurred, of which the profession has never been advised, and that it has often been the cause of injury, and even death, when its agency has not been recognised, especially in the hands of those who were prepared to witness only its salutary operation. A fatal case is reported in a late number of one of our medical periodicals as descriptive of a form of congestive fever, which it is alleged the quinine failed to cure, but which to my mind it most clearly produced. The valuable paper by Prof. S. H. Dickson, so often referred to here, contains some valuable hints touching this subject; in one paragraph he says: 'Look for its injurious operation upon the human system as displayed in a set of phenomena closely resembling the series of symptoms which characterize the very class of fevers in which it is most clearly indicated.' And who can doubt, after knowing the doses in which the unfortunate Dr. Bazire employed it, that the 'power of his remedy' produced the very state of things which he blindly persisted in believing it would relieve if only given in sufficient quantities, until, with this wild though honest conviction, he doubled for himself the already fatal dose? Perhaps in other places as well as in Martainville (Dr. Brazire's residence) we would have fewer cases of 'malignant fevers' if quinine was used with more moderation.

"Under the impression that quinine is a poison, and one capable of producing death when given in over doses, and that it is frequently prescribed nowadays in quantities calculated to produce this result, and knowing that it is not regarded by a large portion of the profession in the same light, I am induced to offer these observations and reflections in regard to it—not with the view or desire, however, of lessening its judicious employment; for under a careful and proper administration of it, I know of no single remedy half so valuable to the practitioner of medicine as that of quinine, but like other remedies capable of effecting much good, it may also be made the instrument of incalculable mischief. And if this article should serve to abate the boldness, not to say criminal recklessness, of one 'Dr. Bazire,' I shall feel that I am amply paid for the time and trouble its preparation has cost me."

